# Radial Nano-Heterojunctions Consisting of CdS Nanorods Wrapped by 2D CN:PDI Polymer with Deep HOMO for Photo-Oxidative Water Splitting, Dye Degradation and Alcohol Oxidation

**DOI:** 10.3390/nano13091481

**Published:** 2023-04-26

**Authors:** Pawan Kumar, Ehsan Vahidzadeh, Kazi M. Alam, Devika Laishram, Kai Cui, Karthik Shankar

**Affiliations:** 1 Department of Electrical and Computer Engineering, University of Alberta, 9211-116 St., Edmonton, AB T6G 1H9, Canada; 2 Department of Chemical and Petroleum Engineering, University of Calgary, 2500 University Drive NW, Calgary, AB T2N 1N4, Canada; 3 Nanotechnology Research Centre, National Research Council of Canada, Edmonton, AB T6G 2M9, Canada; 4 Department of Chemistry, Indian Institute of Technology Jodhpur, Jodhpur 34201, India

**Keywords:** CdS, carbon nitride, polyimide polymer, surface passivation, pollutant degradation, alcohol oxidation, photocatalysis, water splitting

## Abstract

Solar energy harvesting using semiconductor photocatalysis offers an enticing solution to two of the biggest societal challenges, energy scarcity and environmental pollution. After decades of effort, no photocatalyst exists which can simultaneously meet the demand for excellent absorption, high quantum efficiency and photochemical resilience/durability. While CdS is an excellent photocatalyst for hydrogen evolution, pollutant degradation and organic synthesis, photocorrosion of CdS leads to the deactivation of the catalyst. Surface passivation of CdS with 2D graphitic carbon nitrides (CN) such as g-C_3_N_4_ and C_3_N_5_ has been shown to mitigate the photocorrosion problem but the poor oxidizing power of photogenerated holes in CN limits the utility of this approach for photooxidation reactions. We report the synthesis of exfoliated 2D nanosheets of a modified carbon nitride constituted of tris-s-triazine (C_6_N_7_) linked pyromellitic dianhydride polydiimide (CN:PDI) with a deep oxidative highest occupied molecular orbital (HOMO) position, which ensures sufficient oxidizing power for photogenerated holes in CN. The heterojunction formed by the wrapping of mono-/few layered CN:PDI on CdS nanorods (CdS/CN:PDI) was determined to be an excellent photocatalyst for oxidation reactions including photoelectrochemical water splitting, dye decolorization and the photocatalytic conversion of benzyl alcohol to benzaldehyde. Extensive structural characterization using HR-TEM, Raman, XPS, etc., confirmed wrapping of few-layered CN:PDI on CdS nanorods. The increased photoactivity in CdS/CN:PDI catalyst was ascribed to facile electron transfer from CdS to CN:PDI in comparison to CdS/g-C_3_N_4_, leading to an increased electron density on the surface of the photocatalyst to drive chemical reactions.

## 1. Introduction

The multifaceted applications of capturing solar energy such as the production of clean fuels via hydrogen generation from sunlight-driven water splitting, light-driven catalytic organic synthesis and harmful pollutant degradation have provided an explicit route to minimize the impact on the environment to develop a carbon-neutral green economy [1,2]. Direct solar-to-hydrogen (STH) production using the photoelectrochemical (PEC) process requires a semiconductor photocatalyst that can simultaneously harvest solar energy to generate electron–hole pairs and use those photogenerated carriers to drive water oxidation/reduction reactions [3]. Numerous semiconductor materials such as oxides, mixed oxides, sulfides, nitrides, phosphides, carbon-based materials, fractional oxide and oxynitrides, etc., have been investigated for water splitting [4,5,6,7,8,9,10]. Among them, chalcogenides (and CdS in particular) have shown great promise due to their low-to-moderate bandgaps (2.4 eV for CdS), tunable morphology and favorable band edge positions. However, photocorrosion (deactivation due to the release of toxic Cd^2+^ species) and a less oxidative valence band restricts the practical application of CdS and other chalcogenides [11]. The overall water splitting performance is often curbed by the poor kinetics of the oxygen evolution reaction (OER) that does not synchronize with the hydrogen evolution reaction (HER). To overcome these problems, sacrificial electron donors which are oxidized at a lower oxidation potential and therefore complement OER are used, which is undesirable because of their cost and the limited commercial applications of the generated by-products. The use of a semiconductor with a more oxidative VB (valence band) (e.g., TiO_2_) is a promising approach, but this typically comes at the cost of an increased bandgap and compromised visible light absorption. Therefore, making a heterojunction of strongly visible light absorbing CdS with a deep valence band containing semiconductor might solve the problem. Additionally, surface passivation of CdS by encapsulation with a photoactive protective layer has been demonstrated to minimize photocorrosion and ameliorate photoactivity [12,13]. Graphitic carbon nitride (g-C_3_N_4_; CN) is a promising candidate for surface passivation, heterojunction formation, single atom catalysts, solar cells, pollutant degradation, etc., due to facile dopability, simple protocols for surface functionalization, tunable density of reactive sites and favorable photophysical properties [14,15,16,17,18]. Previous reports demonstrated that coupling morphologically tuned CdS with CN can improve the relative performance of the catalyst due to better charge separation and surface passivation [19,20]. Nonetheless, CdS/g-C_3_N_4_ heterojunctions never achieved practical performance metrics because of colossal inter-sheet charge recombination in π-stacked g-C_3_N_4_ and the unfavorable flow of electrons from g-C_3_N_4_ to CdS in a core–shell structure reducing the availability of electrons on the surface of the catalyst. Fortunately, the band structure of carbon nitride can be manipulated by modification of chemical structure and bonding pattern of heptazine (tris-*s*-triazine; C_6_N_7_) units [21,22]. Recently, we have shown that in a carbon nitride framework with C_3_N_5_ stoichiometry constituted of azo-linked tris-*s*-triazine units, the band gap value can be reduced to 1.76 eV [23]. Shiraishi et al. demonstrated that the introduction of electron-deficient pyromellitic diimide (PDI) units in the g-C_3_N_4_ scaffold (g-C_3_N_4_/PDI) via a thermal condensation reaction between melem and pyromellitic dianhydride (PMDA) can drastically shift the VB position towards a more positive value [24]. Further, by controlling the number of incumbent PDI units, the band structure can be tuned. The g-C_3_N_4_/PDI synthesized via equimolar amounts of melem and PMDA displayed the best performance for oxidation of water to H_2_O_2_. Note that the use of CN incorporating PDI units is extremely rare and we are not aware of any reports on exfoliated two-dimensional nanosheets of CN:PDI polymers. The issue of prodigious charge recombination in bulk CN can be solved by its transformation into few-layered sheets which facilitate better charge separation and ensure a shorter distance that photogenerated carriers need to diffuse to react with surface adsorbates and/or electrolyte ions [25]. Hydrogen bonds are responsible for intra-sheet and inter-sheet potential barriers for charge carriers which in turn reduce carrier mobility and increase the probability of recombination losses [26]. Among various methods of forming 2D nanosheets such as solvent exfoliation, gas templating precursors, alkali-assisted exfoliation and acid-assisted exfoliation, the acid-based approach is captivating because it proceeds with the breaking of hydrogen bonds between strand NH/NH_2_ forming monolayered to few-layered sheets [27,28]. Zhang et al. synthesized a gel of monolayer CN sheets using concentrated HNO_3_ at 80 °C which protonated NH/NH_2_ while nitrate ions oxidized some fraction resulting in the separation of sheets [29]. Interestingly, once the solvent is removed, the sheets restack in a more ordered and periodic fashion to facilitate better charge separation. Making heterojunctions using graphenic 2D semiconductors and inorganic semiconductors such as 1D SnIP, TiO_2_, BiVO_4_, 2D WS_2_, MoS_2_, black phosphorus, etc., has been shown to result in a synergistic improvement in photocatalytic performance [5,9,12,30,31,32,33,34,35]. Considering the deep HOMO level of g-C_3_N_4_/PDI (we denoted an equimolar g-C_3_N_4_/PDI as CN:PDI), we intend to produce a heterojunction of CdS and chemically exfoliated CN:PDI (CdS/CN:PDI) which can achieve a favorable photo-induced electron transfer from the CdS to CN:PDI in the absence of an external bias. The oxidation of benzyl alcohol (BA) to benzaldehyde (BAL) would benefit from an eco-friendly, sunlight-driven reaction due to its industrial relevance in synthetic and medicinal chemistry [36]. Conventional approaches use toxic/hazardous stoichiometric reagents (i.e., chromium (VI) reagents, permanganates, 2-iodoxybenzoic acid, etc.) and hydrogen peroxide for such oxidation [37,38]. The photocatalytic oxidation of benzyl alcohol not only provides an energy-efficient green route, but also works as a sacrificial donor in water splitting complementing the OER half-reaction [39]. ZnS-Ni_x_S_y_, Ni-decorated Zn_0_._5_Cd_0_._5_S, binuclear platinum(II) diphosphite complexes (Ptpop), etc., can derive a proton from the valence band (VB) hole-mediated oxidation of BA which can be coupled to the electron-mediated reduction reaction at the conduction band (CB) to produce hydrogen [40]. Previously, CdS decorated with noble/transition metal or metal oxide particles has been used for photocatalytic oxidative-dehydrogenation of BA to BAL [41,42]. The surface-passivated CdS/CN:PDI can alleviate the issue of photocorrosion and improve performance. Furthermore, the CdS/CN:PDI was also explored for the photocatalytic degradation of methylene blue (MB) and rhodamine B (RhB), and demonstrated excellent decoloration efficiency.

## 2. Materials and Methods

Detailed synthesis and characterization protocols are provided in the Supporting Information.

## 3. Results

**Figure 1:**
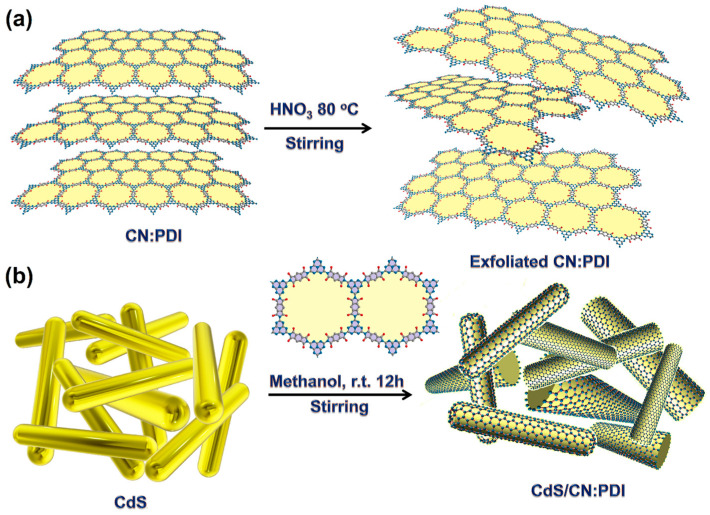
Schematic diagram of the synthesis of (**a**) exfoliated carbon nitride polydiimide (CN:PDI) polymer, (**b**) wrapping of CN:PDI on CdS nanorods.

The CdS nanorods were synthesized via a hydrothermal reaction of CdCl_2_·2.5H_2_O and thiourea in an autoclave as described in the literature [43,44]. Thermal condensation polymerization of dicyandiamide at 550 °C was used for the synthesis of CN containing fused tris-*s*-triazine (heptazine, C_6_N_7_) units [45,46]. Bulk PMDA units incorporated CN (CN:PDI) semiconductor polymer was synthesized by thermal annealing of PMDA and melem (2,5,8-triamino-s-heptazine; C_6_N_7_-(NH_2_)_3_) [47] at 425 °C for 12 h. Since the bulk CN:PDI synthesized using an equimolar mixture of PMDA and melem has been reported to exhibit the highest photoactivity, we used CN:PDI equimolar units containing catalyst throughout this study [24,48]. The bulk CN:PDI was transformed into few-layered CN:PDI by proton-assisted exfoliation using conc. HNO_3_ at 80 °C (Figure 1a) [29]. The protons furnished by HNO_3_ can protonate strand nitrogens (NH/NH_2_) on CN:PDI to form ammonium-type local sub-structure which breaks intersheet hydrogen bonding resulting in the exfoliation of π-stacked sheets [17,49]. Further, strongly oxidizing nitrate ions (NO_3_^−^) partially oxidize sheets promoting mutual repulsion and exfoliation of sheets. The obtained Exf. CN:PDI sheets were washed with water and redispersed in methanol. The CdS/CN:PDI heterojunction in which the CdS nanorod core was enwrapped with CN:PDI sheets was achieved via the mixing of CdS and Exf. CN:PDI suspension in methanol for 12 h. The average size(s) of CdS, Exf. CN:PDI and CdS/CN:PDI were determined to be 190, 270 and 472 nm, respectively, as determined using dynamic light scattering (DLS) (Figure S2). The increased hydrodynamic radius of CdS after coupling with CN:PDI demonstrates that CN:PDI nanosheets successfully wrapped around CdS nanorods in CdS/CN:PDI.

**Figure 2:**
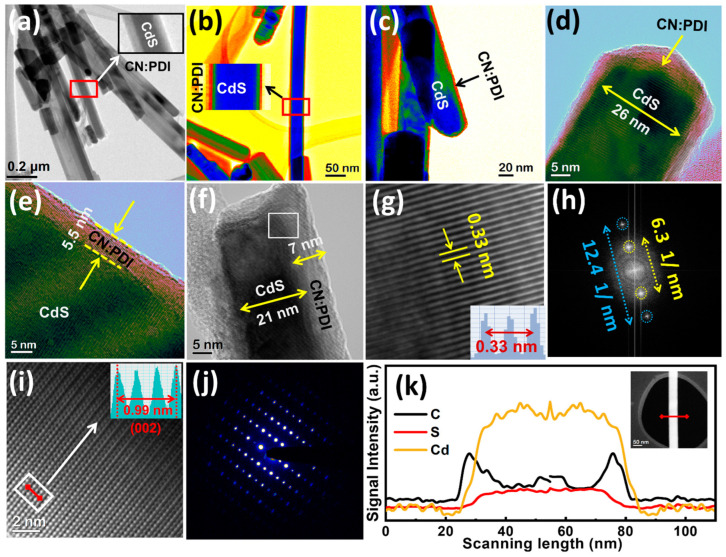
HR-TEM image of CdS/CN:PDI at (**a**) 0.2 µm (**b**) 50 nm (**c**) 20 nm scale bar showing CdS nanorod bundles with CN:PDI coating and HR-TEM images at (**d**–**f**) 5 nm scale bar showing the diameter of CdS and thickness of CN:PDI coating, (**g**) image of the selected area showing lattice fringes at the interface (**h**) FFT of the selected area (**i**) HR-TEM image at 2 nm scale bar showing the atomic column of CdS nanocrystal; inset showing d-spacing between atomic dots. (**j**) SAED pattern of CdS/CN:PDI and (**k**) electron energy-loss spectra (EELS) line scan on CN:PDI showing the distribution of Cd (yellow), S (red) and C (black) along the diameter of CdS/CN:PDI.

The structural attributes of the synthesized samples were determined using high-resolution transmission electron microscopy (HR-TEM) (Figure 2a–h and Figure S1). The HR-TEM images of CdS/CN:PDI at a relatively low magnification (200 nm scale bar) display bundles of CdS nanorods (Figure 2a). The magnified view clearly shows the presence of CN:PDI wrapped around the CdS nanostructure. The color map TEM images of CdS/CN:PDI at 50 and 20 nm scale bars clearly show distinct wrapping of CN:PDI on CdS nanorods (Figure 2b,c). The high-resolution TEM image at a 5 nm scale bar shows that the diameter of the CdS nanorods was between 21 and 26 nm with a 5–7 nm coating of CN:PDI (Figure 2d–f). The CdS lattice fringes with a *d* spacing of 0.33 nm were clearly evident in TEM images (Figure 2g). It is interesting to note that CdS and CN:PDI have a similar 0.33 nm lattice spacing, and thus a clear distinction between CN:PDI and CdS interface lattice planes at their interface is not possible. However, identical lattice fringes for a sparse and dense area in TEM images indicate the highly crystalline nature of wrapped CN:PDI. FFT of the interface between the sparse layer of CN:PDI and CdS demonstrate two sharp spots at 6.3 1/nm (0.32 nm) due to 002 planes and 12.4 1/nm (0.16 nm) due to overlapped planes (Figure 2h). Further, the TEM image at 600x magnification and 2 nm scale bar shows an atomic column of CdS, revealing the highly ordered and crystalline nature of CdS nanorods (Figure 2i) [50]. The calculated interplanar distance of these columns was determined to be 0.33 nm, and was assigned to 002 planes of CdS. The selected area electron diffraction (SAED) pattern displayed bright diffraction spots due to (002), (112) and (110) demonstrating the excellent crystallinity of CdS nanorods (Figure 2j) [51]. To prove that CN:PDI was wrapped all around the nanorods, electron energy loss spectroscopy (EELS) to measure the intensity of C *K*-edges, Cd *M-*edge and S *L*-edges energy loss was used (Figure 2k). The EELS line scan across the diameter of nanorods clearly shows a strong distribution of Cd and S due to the presence of CdS core while a weak signal of carbon with a relatively higher intensity at edge validates the presence of CN:PDI wrapped around CdS nanorods.

**Figure 3:**
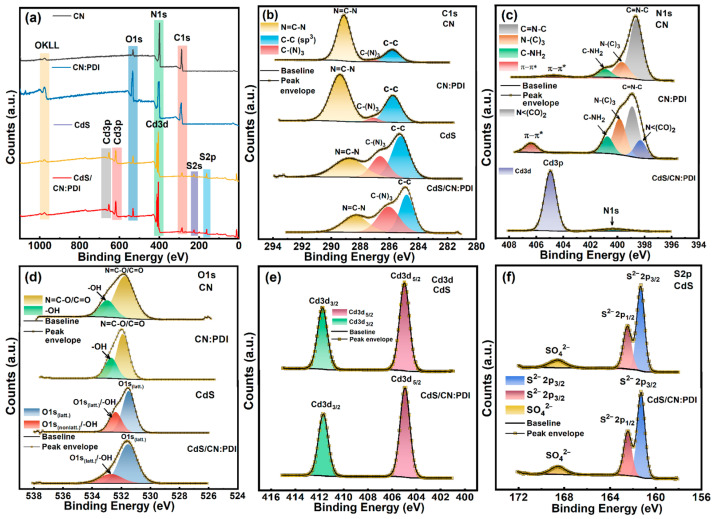
(**a**) XPS survey scan of CN (black), CN:PDI (blue), CdS (yellow), CdS/CN:PDI (red) and Core-level HR-XPS spectra of CN, CN:PDI, CdS, and CdS/CN:PDI in (**b**) C1s (**c**) N1s (**d**) O1s (**e**) Cd3d and (**f**) S2p region.

The surface chemical nature and binding energies of constituting elements were determined using X-ray photoelectron spectroscopy (XPS) (Figure 3). The low-resolution XPS survey of materials contained all the core level and the sub-core-level peaks of constituent elements in CN (C1s, N1s, O1s, OKLL), CN:PDI (C1s, N1s, O1s, OKLL), CdS (Cd3d, S2p, Cd3p) and CdS/CN:PDI (C1s, N1s, O1s, Cd3d, S2p, Cd3p, OKLL) (Figure 3a). The high O1s peak intensity in CN:PDI XPS suggests incorporation of C=O containing PMDA units. The XPS quantification considering the relative sensitivity factor is provided in Table S1. The high-resolution (HR) XPS spectra of CN in the C1s region was deconvoluted into three peak components centered at binding energy values of 284.8, 286.2 and 288.3 eV. The peak at BE 284.8 eV originated from sp^3^ hybridized C-C turbostratic adventitious carbons while XPS peaks at 286.2 and 288.3 eV were assigned to tertiary (N-(C)_3_) and secondary (N-C=N) carbons of sp^2^ hybridized heptazine nucleus (C_6_N_7_) composing the carbon nitride framework (Figure 3b) [30,52]. The Exf. CN:PDI polymer sheets synthesized in this work also demonstrated three peak components in the C1s region located at the same BE value due to sp^3^ hybridized (C-C), sp^2^ hybridized tertiary (N-(C)_3_) and secondary (N-C=N) carbons. From the C1s XPS spectra of Exf. CN:PDI, it can be seen that the intensity of the sp^3^ hybridized (C-C) peak component was increased which was attributed to the contribution of carbons present in pyromellitic diimide (PDI) units. The CdS showed three C1s peaks at 284.8 (C-C), 286.2 ((N-(C)_3_) and 288.2 (N-C=N carbons) eV due to residual organic precursor/their hydrothermal products used in the hydrothermal synthesis. After wrapping CN:PDI around CdS nanorods, the peak intensities of (N-(C)_3_) and (N-C=N carbons) increased due to the contribution from CN:PDI. The deconvoluted core level HR-XPS spectra of CN in the N1s region displayed three peak components at BE ≈ 398.6, 399.7, and 400.9 eV (Figure 3c). The XPS peak components at 398.6 and 399.7 eV arose from the secondary C=N–C and tertiary N–(C)_3_ nitrogens of N-linked heptazine (C_6_N_7_) in the CN scaffold while another relatively small peak component centered at 400.96 eV was observed due to residual sp^3^ hybridized primary nitrogens present at the edge of the sheets (–NH_2_/NH) (Figure 3c) [53]. A weak signal at the BE ≈ 404.75 eV was observed due to π-π* transition in the conjugated CN system. Besides these three peak components, an additional peak at 398.3 eV due to polyimide bond nitrogens (N < (CO)_2_) was also observed in the XPS spectra of CN:PDI. Furthermore, the π-π* transition peak for CN:PDI was observed at a relatively high binding energy (406.42 eV) along with increased intensity. The increased binding energy occured due to the electron-withdrawing effect of electron-deficient PMDA units which increased the bandgap due to band edge shifting resulting in high energy π-π* transition in harmony with the previous reports [54]. Further, the addition of aromatic rings containing PMDA units in the CN:PDI structure enhanced the degree of conjugation which resulted in increased peak intensity. Unfortunately, the N1s signals in XPS spectra of CdS/CN:PDI were suppressed due to an intense Cd3p peak at 404.90 eV coinciding in the same region, and only a very small N1s peak was observed. The peak deconvolution of the O1s peak of CN revealed two peak components at 531.71 and 533.07 eV (Figure 3d). The peaks at 531.71 eV originated from the C=O/N-C-O oxygens in the residual uncondensed CN structure, while the shoulder peak at 533.05 eV was assigned to -OH of adventitious oxygen/moisture (Figure 3c). The C=O/N-C-O oxygens and -OH peaks were also present in HRXPS of CN:PDI, and C=O/N-C-O peaks were shifted slightly toward high BE due to the electron withdrawing effect of PMDA [55]. The O1s spectra of CdS also show two peaks at 531.6 and 532.8 eV due to surface-oxidized oxygens present in SO_4_^2−^/O^2−^ state oxygens and surface-adsorbed -OH oxygens [56]. The HR-XPS spectra of CdS and CdS/CN:PDI in the Cd3d region showed two well-separated XPS peaks at 404.9 and 411.7 eV assigned to Cd3d_5/2_ and Cd3d_3/2_ peak components of Cd present in +2 oxidation state of CdS (Figure 3e) [12]. Furthermore, core-level HR-XPS spectra of CdS and CdS/CN:PDI in the S2p region demonstrated three deconvoluted peaks located at BE values 161.3, 162.5 and 168.5 eV (Figure 3f). The major strong peak at 161.3 eV and adjacent shoulder peak at 162.5 eV were assigned to S2p_3/2_ and S2p_1/2_ peak components of sulfide (S^2−^) ions present in the CdS crystal structure [57] while a less intense peak at 168.5 eV originated from surface-oxidized S atoms present in sulfate form (SO_4_^2−^) [58]. The BE value for Cd3d and S2p peak remains unchanged after the wrapping of CN:PDI suggests the absence of any ionic interaction and substantiating the presence of a pure Van der Waals interaction between CdS and CN:PDI.

**Figure 4:**
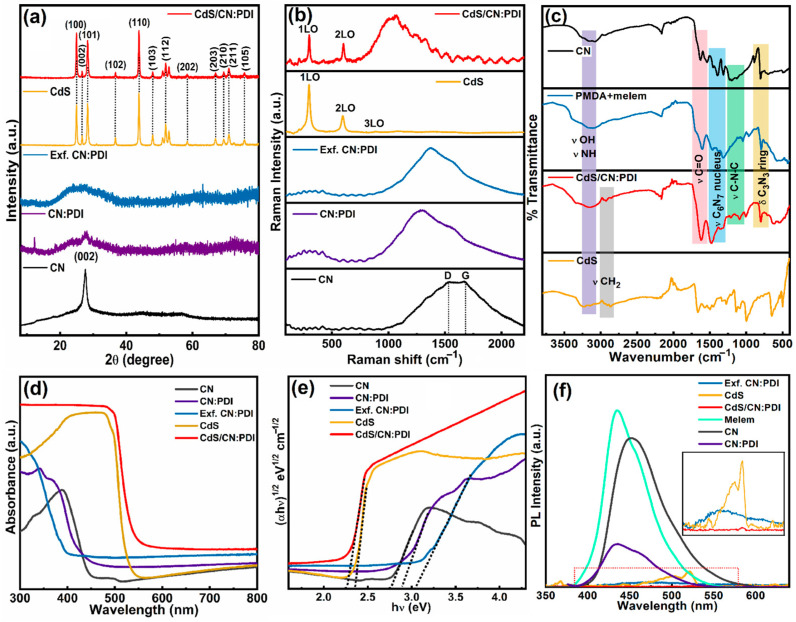
(**a**) XRD diffraction pattern, (**b**) Raman spectra, (**c**) FTIR (**d**) DR-UV–Vis spectra (**e**) Tauc plot and (**f**) steady-state PL spectra of CN (black), CN:PDI (violet), Exf. CN:PDI (blue), CdS (yellow) and CdS/CN:PDI (red).

X-ray diffraction (XRD) was used for the determination of the crystalline nature and the periodic structure of the materials (Figure 4a). The X-ray diffractogram of CN displays two peaks at 2θ values of 27.6° and 13.5° attributed to (002) and (100) planes, respectively, of CN stacked in graphitic structure [59]. The (002) XRD peak with a 0.32 nm interplanar *d* spacing originated from the interplanar sheets stacking, while a relatively weak (100) peak at 13.5° with 0.68 nm interplanar distance was assigned to in-plane packing of C_6_N_7_ units in CN network. The bulk CN:PDI displayed weak peaks at 27.7°, 18.5° and 12.3°. The first peak at 27.7° occurred due to π-stacking, while two peaks at 18.5° and 12.3° originated due to melem-pyromellitic dianhydride polydiimide structure as reported in the literature [60]. In comparison to CN, the peak intensity corresponding to the (002) plane was much lower for the CN:PDI which suggests decreased π stacking and crystallinity due to the incorporation of PMDA units. After the transformation of bulk CN:PDI into exfoliated sheets, the (002) peak becomes broadened, which might be due to distortion of π stacking and breaking of hydrogen bonds via HNO_3_ treatment. The XRD patterns of CdS displayed diffraction peaks at 24.95° (100), 26.65° (002), 28.33° (101), 36.69° (102), 43.84° (110), 48.01 (103), 51.89 (112), 58.47 (202), 66.93 (203), 69.39 (210), 70.99° (211) and 75.65° (105) indexed to hexagonal wurtzite crystal structure (JCPDS card no. 41-1049) [61]. The XRD diffractogram of CdS/CN:PDI heterojunction shows all the peaks corresponding to CdS. The absence of any peak of CN:PDI in CdS/CN:PDI indicates that the wrapped CN:PDI layer is too thin to produce any detectable signal.

To gain more insight into the nature of chemical functionalities, Raman spectra of CN, bulk CN:PDI, Exf. CN:PDI, CdS and CdS/CN:PDI were collected under 632 nm excitation wavelength and 10 mW cm^−2^ laser power (Figure 4b). The Raman spectra of CN displayed two vibrational peaks at 1530 cm^−1^ and 1670 cm^−1^ assigned to the D and G bands of the graphitic structure of carbon nitride [62]. The D band refers to defects originating from the out-of-plane vibration of sp^3^ hybridized carbons bonded to nitrogens (C-N) while in-plane vibration of sp^2^ hybridized carbons in graphitic heptazine network gives rise to the G band (graphitic). Due to variation in the C-N bond angle in sp^2^ hybridized heptazine moieties, the carbon nitride structure deviates from planarity. The out-of-plane vibration of these sp^2^ C-N’s in the N-linked heptazine structure produces the D band Raman mode [16]. Contrarily to CN, the peak intensity of the G band was decreased for CN:PDI due to the addition of sp^3^ hybridized carbons from PMDA units in the CN:PDI network. Furthermore, the decreased G band intensity was in good agreement with XRD results suggesting decreased π stacking and graphitic structure. After the transformation of bulk CN:PDI into Exf. CN:PDI, the Raman signal does not change, signifying that the chemical structure of CN:PDI remains intact during the exfoliation step. The Raman spectra of CdS nanorods displayed the signature longitudinal optical (LO) mode peak at 298 cm^−1^ and corresponding second-order (2LO) and third-order (3LO) features at 593 and 885 cm^−1^, respectively [63]. The CdS/CN:PDI heterostructure obtained by the wrapping of CN:PDI on CdS nanorods displayed all the signature peaks of CdS. Furthermore, a Raman peak assigned to the combined vibration of the D + G band was also observed, indicating the presence of CN:PDI on the surface of CdS nanorods.

The nature of surface chemical groups responsible for distinct vibrational features in the infrared region was determined using Fourier transform infrared (FTIR) spectroscopy (Figure 4d). The characteristic FTIR vibration peak at 802 cm^−1^ in the FTIR spectra of bulk CN was observed due to the bending vibration of the triazine (C_3_N_3_) ring in the tris-*s*-triazine (C_6_N_7_) nucleus [64]. The FTIR peaks in the 1035–1512 cm^−1^ region were assigned to the stretching vibrations of the triazine ring [65]. The bending of surface-adsorbed H_2_O and stretching of C=O (δ_H2O_, ν_C=O_) produced an FTIR band between 1523 and 1635 cm^−1^. A broad vibrational band at 3160 cm^−1^ originated due to the stretching vibration of terminal/strand –NH_2_/NH (ν_N–H_) and –OH (ν_O–H_). The FTIR spectra of CN:PDI showed all the signature peaks of the heptazine nucleus containing carbon nitride framework. Additionally, the peak at 1610 cm^−1^ due to C=O stretch was much more intense in comparison to carbon nitride, confirming the presence of PMDA unit in CN [66]. Notably, the vibration peak for CNPDI C=O bond was observed at low wavenumbers because of inclusion in an aromatic conjugated network. The FTIR spectra of CdS, along with the main peak of Cd-S vibration at 648 cm^−1^, demonstrated numerous peaks at 987, 1130, 1265, 1516 and 1670 cm^−1^ due to the residual organic moieties/precursor. In CdS/CN:PDI precursor composite, all the peaks relevant to CN:PDI were present at almost equal intensity, suggesting the contribution of signals from a surface-coated CN:PDI.

The UV–Vis absorption spectra of bulk CN displayed a peak maximum at 388 nm within a broad absorption band extending up to 450 nm due to band-to-band transition between the HOMO constituted of N2p orbitals and the lowest unoccupied molecular orbital (LUMO) constituted of C2p orbital (Figure 4d). The bulk CN:PDI displayed a broad UV–Vis absorption band consisting of two peaks with a band edge extending to 430 nm [67]. Compared to bulk CN, the absorption edge of CN:PDI was observed at a lower wavelength, suggesting a slightly wider bandgap due to the introduction of electron-withdrawing PMDA units. A small absorption peak around 342 nm was attributed to π→π* transition while a relatively intense absorption band at 370 nm was assigned to n→π* transition in CN:PDI framework [68]. After the transformation of bulk CN:PDI into exfoliated sheets, the absorption profile of Exf. CN:PDI was blue-shifted into the UV region with an absorption edge at 394 nm due to reduced electronic interaction and confinement effect. Previous reports and theoretical calculations also suggest a wider electronic bandgap as the number of stacked sheets is reduced (usually below four), suggesting successful exfoliation of CN:PDI sheets. The CdS nanorods displayed a distinct absorption extending to 530 nm followed by a steep band edge due to the electronic VB to CB transition. In CdS/CN:PDI heterojunction, after wrapping of CN:PDI, the absorption edge was slightly red-shifted, which might be due to synergistic electronic interaction between CdS and CN:PDI. Additionally, the optical band gap of all the materials was determined from the Tauc plot. The band gap was calculated by plotting a graph between (*α*h*ν*)^1/2^ vs. photon energy (*hν*) followed by extrapolation of the linear tangent on the *x*-axis, which provides the value of bandgap, where *α* is the absorption coefficient, *h* is plank constant and *ν* is the frequency of light (Figure 4e). The calculated band gaps of CN, bulk CN:PDI, Exf. CN:PDI, CdS and CdS/CN:PDI were 2.74, 2.88, 3.04, 2.35 and 2.26 eV, respectively.

The nature of the charge carrier recombination was examined using photoluminescence (PL) spectroscopy under a 365 nm excitation light source (Figure 4f). The PL spectra of melem displayed an intense and broad PL peak centered at 434 nm originating from the intense charge recombination between HOMO-LUMO molecular orbitals of stacked C_6_N_7_ moieties [18,69]. For the bulk carbon nitride, the PL peak was shifted toward a longer wavelength at 450 nm due to the decreased bandgap of tertiary nitrogen-linked heptazine units [70]. The PL intensity was significantly decreased for the bulk CN:PDI polymer due to the introduction of PMDA units which facilitates better charge separation between the melem and pyromellitic polydiimide motif reducing the rate of radiative recombination. A careful evaluation of CN:PDI’s PL band demonstrates that the broad PL band was constituted of two merged/overlapping peaks that likely originate from the contribution of melem and PMDA units. The PL peak intensity for Exf. CN:PDI was dramatically decreased after the exfoliation due to reduced localized intersheet charge carrier recombination in conjugated sheets. The CdS nanorods demonstrated a weak emission peak at 521 nm due to the intrinsic band-to-band recombination of charge carriers [71]. The PL peak intensity for CdS/CN:PDI heterojunction almost disappeared due to better charge separation of photogenerated charge carriers in the heterojunction.

Photoelectrochemical (PEC) water splitting

**Figure 5:**
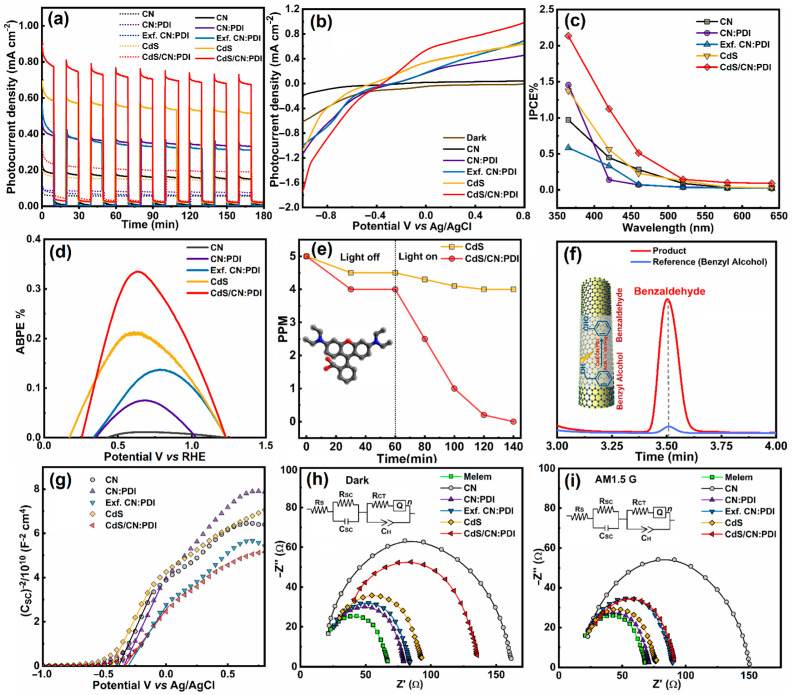
(**a**) Photocurrent vs. time (*i-t*) plot showing photoresponse during light On–Off cycle at +0.6 V under solar-simulated AM1.5G light irradiation without filter (100 mW cm^−2^) with and AM1.5G light irradiation with a 420 nm cut-off filter. (**b**) LSV showing photocurrent density with respect to the applied voltage and (**c**) IPCE% vs. wavelength action spectra under 365, 420, 460, 520, 580, 640 nm wavelength LEDs (21.0 mW cm^−2^). (**d**) ABPE% showing maximum photoconversion efficiency (PCE%) under AM1.5G light irradiation without filter (100 mW cm^−2^) for CN, CN:PDI, Exf. CN:PDI, CdS, CdS/CN:PDI. (**e**) Photocatalytic degradation of RhB using CdS (yellow), CdS/CN:PDI (red) under AM1.5G light, (**f**) HPLC chromatogram of pure BA (blue) and the reaction product of photooxidation of BA showing the peak of BAL (red) using CdS/CN:PDI as a catalyst. (**g**) Mott–Schottky plot showing flat band potential. (**h**) EIS Nyquist plot under dark and (**i**) under AM1.5G irradiation. Inset showing Randles equivalent circuit. All the measurements were performed in 0.1 M Na_2_SO_4_ solution at a scan rate of 0.1 mV/s. Color: CN (black), CN:PDI (violet), Exf. CN:PDI (blue), CdS (yellow), CdS/CN:PDI (red).

The PEC performance of the synthesized materials was evaluated by using each of them as a photoanode for sunlight-driven water splitting (Figure 5). In a three-electrode setup, films of the synthesized material deposited on fluorine-doped tin oxide (FTO) coated glass substrate were used as the anode, while Pt and Ag/AgCl electrodes were used as counter and reference electrodes, respectively. The photocurrent density as a function of time (*J-t*) during the light On–Off cycle was determined at +0.6 V vs. Ag/AgCl (thermodynamic oxidation potential of water +1.23 vs. RHE) under AM1.5G one-sun simulated sunlight (Figure 5a). The photocurrent density of pristine CN was only 0.17 mA cm^−2^, while for bulk CN:PDI, the photocurrent increased up to 0.36 mA cm^−2^ under unfiltered one-sun illumination. Despite the increased bandgap of CN:PDI (2.88 eV) in comparison to CN (2.74 eV), the increased value of photocurrent can be explained based on a deeper HOMO level in CN:PDI due to the introduction of electron-deficient PMDA moieties [72]. The photocurrent density slightly decreased after the transformation of bulk CN:PDI into exfoliated CN:PDI (0.34 mA cm^−2^), which might be due to the increased band gap of Exf. CN:PDI. Pristine CdS displayed a photocurrent value of 0.56 mA cm^−2^ which was further increased after wrapping of CN:PDI in CdS/CN:PDI heterojunction, reaching the highest value of 0.74 mA cm^−2^. The increased photocurrent density after wrapping with CN:PDI occurred because of the surface passivation of CdS nanorods and improved hole extraction. An identical photocurrent pattern was observed under simulated sunlight when a 420 nm ultraviolet (UV) cut-off filter was used (Figures S3–S7). Linear sweep voltammograms (LSV) showing the change in photocurrent density as a function of applied bias were also measured in aqueous sodium sulfate solution in the range of −1.0 to +0.8 V vs. Ag/AgCl, which shows a substantial open circuit potential for all the samples suggesting the efficient transfer of photogenerated holes from the photoanode to the electrolyte facilitating water splitting reactions (Figure 5b). The overall power conversion efficiency (PCE) values under unfiltered AM1.5G radiation calculated from applied bias photoconversion efficiency (ABPE) vs. potential graphs were determined to be 0.011, 0.074, 0.137, 0.209 and 0.335% for CN, bulk CN:PDI, Exf. CN:PDI and CdS/CN:PDI, respectively.

Photocatalytic dye degradation

Encouraged by the increased photocurrent response of CN:PDI-based materials in water splitting, we employed these materials as catalysts for visible-light-driven photodegradation of rhodamine B (RhB) and methylene blue (MB). We chose RhB and MB dyes as model pollutants since they represent anionic and cationic dyes with a distinct HOMO-LUMO and oxidation/reduction potential. Therefore, materials that can degrade these two model pollutants are likely to be successful in photo-oxidizing other pollutants as well. The photocatalytic degradation of RhB and MB (5 ppm each) was performed under UV filtered simulated sunlight (>420 nm) using 20 mg catalysts and the reaction kinetics were determined with the help of UV–Vis measurements. The results of photocatalytic dye degradation are shown in Figure 5e and Figure S9 in Supporting Information. Before irradiation, the reaction mixture was stirred in the dark to achieve adsorption–desorption equilibrium and to ensure true photocatalytic degradation. From the graphs, it can be seen that CN:PDI displayed excellent photocatalytic RhB and MB degradation performance in comparison to bulk CN (Figure S8). The increased photocatalytic performance occurred due to the more oxidative HOMO (+2.94 V vs. NHE) of CN:PDI. Pristine CdS displayed sluggish dye degradation performance (gold-colored curve in Figure 5e). However, after wrapping CN:PDI around CdS nanorods, the dye degradation performance of CdS/CN:PDI was increased, reaching almost complete degradation within 60–80 min. The improved performance of CdS using CN:PDI wrapping was attributed to the synergistically enhanced separation of photogenerated charge in CdS/CN:PDI heterojunction photocatalyst.

Photocatalytic oxidation of benzyl alcohol to benzaldehyde

The application scope of the developed CN:PDI materials in catalyzing organic reactions under solar light was studied using the oxidation of benzyl alcohol (BA) to benzaldehyde (BAL) as a model catalytic reaction (Figure 5). The oxidation of BA to BAL was carried out using acetonitrile as a solvent and molecular oxygen in the air as oxidants under simulated sunlight at room temperature. The progress of the reaction at regular intervals was monitored by thin-layer chromatography on a silica gel-coated aluminum plate. After the reaction, the product was separated from the catalyst using a syringe filter and analyzed using HPLC equipped with ultraviolet (UV) and refractive index (RI) detectors. To confirm the oxidation of BA to BAL, the reaction products were analyzed using an electrospray ionization (ESI) mass spectrometry column to obtain a fragmentation pattern. After 12 h, using a 5 mol% CdS/CN:PDI catalyst, the yield of BAL was 90%, while under identical conditions, pristine CN, CN:PDI, and CdS displayed negligible performance (Figure 5f). No reaction product was observed under dark conditions, validating the reaction to be truly photon-driven.

Mechanism

**Figure 6:**
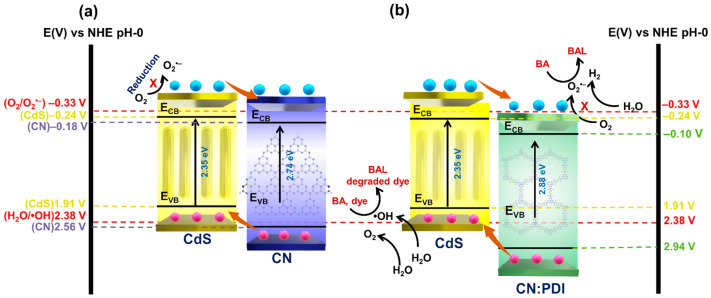
The band diagram displays the flow of electrons in (**a**) CdS/CN (**b**) CdS/CN:PDI.

To explain the mechanism of enhanced photocatalytic performance, the band structure of the synthesized catalysts was determined (Figure 6). The Mott–Schottky plot demonstrates that all the materials exhibit *n*-type conduction (Figure 5g). Further, the flat band potential values of CN, bulk CN:PDI, Exf. CN:PDI, CdS and CdS/CN:PDI in aqueous sodium sulfate electrolyte were determined to be −0.38, −0.33, −0.30, −0.44 and −0.34 V vs. Ag/AgCl, respectively. Since all materials show *n*-type conduction, the Fermi level remains close to the CB and the obtained flat band potential may be considered to be the CB/LUMO position. Further, the obtained CB position vs. Ag/AgCl was converted to the NHE scale and determined to be −0.18, −0.13, −0.10, −0.24, and −0.14 V vs. NHE at pH-0. The calculated band gaps of CN, bulk CN:PDI, Exf. CN:PDI, CdS and CdS/CN:PDI were 2.74, 2.88, 3.04, 2.35 and 2.26 eV, respectively, as previously determined from Tauc plots. Considering these values, the VB/HOMO positions for CN, bulk CN:PDI, Exf. CN:PDI, CdS and CdS/CN:PDI were determined to be 2.56, 2.75, 2.94, 1.91 and 2.12 eV, respectively. The HOMO level of CN became even more positive after the introduction of PMDA (2.75 V vs. NHE) resulting in a HOMO level of 2.94 V vs. NHE after exfoliation. A schematic illustration of the band structure at the photoanode-electrolyte interface under positive applied bias is shown in Figure S8. Interfacial recombination and photocorrosion are huge problems in CdS–electrolyte junctions (Figure S8b). In the presence of the CN:PDI passivating shell around CdS nanorods, CdS is not in direct contact with electrolyte limiting photocorrosion. Further, the cascaded band structure (Figure S8a) mitigates interfacial recombination. As per the thermodynamics for the splitting of water, the CB of semiconductor materials should be more negative than the reduction potential of water (+0.0 V vs. NHE for H^+^/H_2_) while the VB should be more positive than the oxidation potential of water (+1.23 vs. NHE for H_2_O/O_2_). Due to the deep HOMO of CN:PDI, it can split water more easily in comparison to CN and CdS and hence wrapping of CN:PDI around CdS nanorods facilitates better water oxidation [73,74]. The Nyquist plot calculated via electrochemical impedance spectroscopy (EIS) shows the charge transfer resistance (R_CT_) of bulk, and Exf. CN:PDI is much lower than CN as evidenced by a small semicircle in the EIS plot. This decreased R_CT_ in the dark as well as under illumination supports more facile transfer of holes to electrolyte for CN containing PDI units (Figure 5h–i and Table S2). After wrapping of CN:PDI on CdS, the charge transfer resistance increases because CN:PDI interferes with direct charge transfer between electrolyte and CdS. However, under light irradiation, the CdS/CN:PDI heterojunction shows a large decrease in R_CT_, suggesting the system works effectively as a heterojunction to transfer photogenerated holes to the electrolyte.

The photocatalytic degradation of dye proceeds via the generation of electron–hole pairs which react with water and oxygen to generate •OH and superoxide anion O_2_^•−^ radicals. These radicals are the primary species responsible for the degradation of dye molecules due to their extreme reactivity and oxidative power. For the facile generation of •OH radicals, the VB of semiconductors should be more positive than the redox potential essential to generate the •OH radicals from water (+2.38 V vs. NHE •OH/H_2_O). Similarly, to generate O_2_^•−^ radicals, the CB of the catalyst should be more negative than the oxygen reduction potential (–0.33 V vs. NHE; O_2_^•−^/O_2_) [75]. Except for CdS, each catalyst has a more positive VB to generate •OH radicals. In composite, low observed VB position occurred due to the cumulative average of the band gap. Further, no catalyst has a more negative reduction potential to generate O_2_^•−^ radicals. This suggests •OH radicals should be the main species involved in the dye degradation. Among these materials, Exf. CN:PDI has the most oxidative VB; thus, the coating of CN:PDI on CdS has synergistic action and facilitates better charge separation. The comparison (Table S3) demonstrates CdS/CN:PD can reach a favorable degradation efficiency within 140 min.

**Figure 7:**
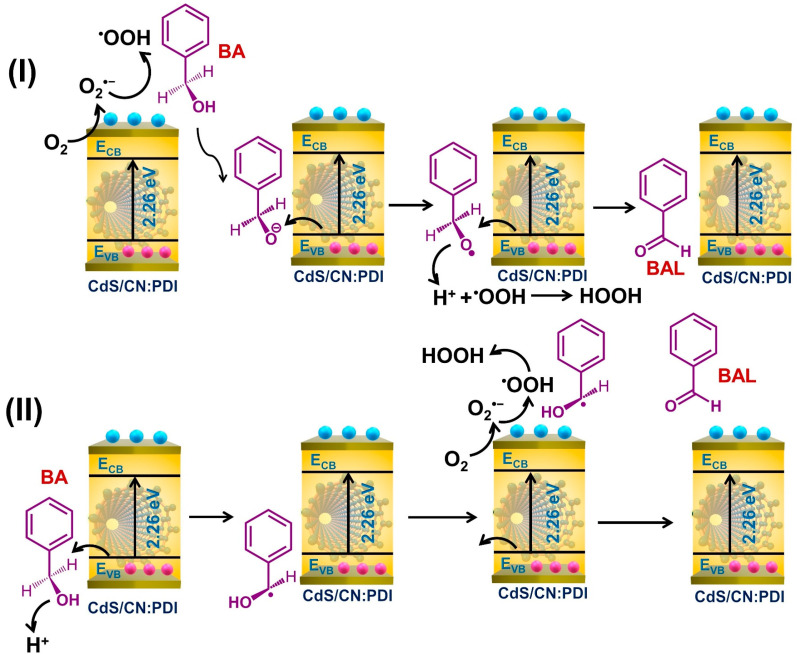
Mechanism of photo-oxidation of benzyl alcohol to benzaldehyde using CdS/CN:PDI via route I and route II.

The photooxidation of BA to BAL is triggered by the absorption of photons by CdS/CN:PDI and the generation of electron–hole pairs. The generated electrons and holes can initiate the oxidation of BA to BAL. Depending upon the first involvement of electrons or holes, the oxidation of BA to BAL can occur via two routes: (I) e^−^/O_2_^•−^ initiated O–H cleavage, and (II) h^+^ initiated C–H cleavage as shown in Figure 7 [76,77,78]. In route I, the generated electrons react with oxygen to generate O_2_^•−^ radical. The O_2_^•−^ radicals are very reactive and abstract a proton from the surface-adsorbed BA forming hydroperoxyl radical (•OOH) and benzyloxide anion (PhCH_2_O:^−^). These Ph(OH)CH:^−^ anions react with holes in the VB and form benzyloxide radical (PhCH_2_O•) which further reacts with the hole and •OOH radical to finally produce BAL and H_2_O_2_. In route II, the holes react with BA resulting in the abstraction of a proton by cleavage of the C-H bond to form a radical (Ph(OH)CH•). These radicals react with superoxide anion radical (O_2_^•−^) generated on the CB of the materials to form hydroperoxyl radical (•OOH) and BAL by proton abstraction. The •OOH radical reacts with protons and electrons to form H_2_O_2_. The comparison of CdS/CN:PDI photocatalytic activity for BA to BAL conversion with previously reported state-of-the-art catalysts are listed in Table S3 which depicts CdS/CN:PDI possess a promising performance (Table S4).

## 4. Conclusions

In conclusion, we have devised a facile approach to passivate the surface of CdS nanorods using monolayer/few layered CN:PDI polymer. The 2D monolayer to few-layered sheets of CN:PDI were synthesized by proton-assisted exfoliation using HNO_3_. The self-assembly of π conjugated nanosheets around the CdS nanorods improves the charge carrier extraction from the nanorods. In contrast to a conventional heterojunction, the deep HOMO level of CN:PDI establishes a staggered heterojunction, leading to better electron extraction at the surface of CN:PDI polymer. The deep HOMO level of CN:PDI also improves the oxidizing power of carbon nitride and its heterojunction with CdS. The CdS:PDI heterojunction demonstrated significantly increased PEC water splitting and dye degradation performance. Additionally, the developed material exhibited an excellent conversion rate for the transformation of benzyl alcohol to benzaldehyde.

## Data Availability

The data presented in the manuscript can be obtained from the corresponding author on a valid request.

## Supplementary Materials

The following supporting information can be downloaded at: https://www.mdpi.com/article/10.3390/nano13091481/s1, Experimental details; Synthesis and Characterization, PEC water splitting, dye degradation, benzyl alcohol to benzaldehyde oxidation, calculations of efficiencies; HR-TEM images, DLS particle size distribution, PEC water splitting (LSV and *i-t* curve under AM1.5G irradiation), Interfacial band-diagram for PEC water splitting, Photocatalytic dye degradation experiments (Figures S1–S9). Table S1: The elemental composition of materials determined using XPS analysis; Table S2: The EIS Nyquist plot fitting parameters to extract various fitting elements of the equivalent circuit under dark and light (AM1.5G) conditions; Table S3: Comparison of photocatalytic activity of CdS/CN:PDI for dye degradation with state-of-the-art catalysts; Table S4: Comparison of photocatalytic activity of CdS/CN:PDI for benzyl alcohol oxidation with state-of-the-art catalysts. References [79,80,81,82,83,84,85,86,87,88,89,90,91,92,93,94,95,96,97,98,99,100,101,102,103,104,105,106,107,108,109,110,111,112,113] are cited in the Supplementary Materials.
