# Supplementary material for: Radial Nano-Heterojunctions Consisting of CdS Nanorods Wrapped by 2D CN:PDI Polymer with Deep HOMO for Photo-Oxidative Water Splitting, Dye Degradation and Alcohol Oxidation
Source: Nanomaterials (Basel). 2023 Apr 26;13(9):1481. doi: 10.3390/nano13091481 (PMC10180281; doi:10.3390/nano13091481)
Supplement: Supplementary file 1 [file nanomaterials-13-01481-s001.zip › nanomaterials-2335896-supplementary.pdf]

SUPPORTING INFORMATION

Contents

1. Experimental section .... **Page S2**
1.1 Chemical reagents .... **Page S2**
2. Synthesis of photocatalytic materials .... **Page S3**
2.1 Synthesis of bulk graphitic carbon nitride .... **Page S3**
2.2 Synthesis of bulk carbon nitride polydiimide polymer (CN:PDI) .... **Page S3**
2.3 Synthesis of exfoliated carbon nitride polydiimide polymer (Exf. CN:PDI)................ **Page S3**
2.4 Synthesis of CdS nanorods .... **Page S4**
2.5 CN:PDI wrapped CdS Vander Waals heterostructure (CdS/CN:PDI) .... **Page S4**
3. Physicochemical characterization/measurement .... **Page S5**
4. Calculation of Applied bias photon-to-current efficiency (ABPE)............................ **Page S6**
5. Electrochemical impedance spectroscopy.. **Page S6**

Figures

Figure S1. HR-TEM images and SAED pattern of CdS/CN:PDI...................................... **Page S7**
Figure S2. Particle size distribution of CdS, Exf. CN:PDI and CdS/CN:PDI .... **Page S8**
Figure S3. LSV of CN under AM1.5G and photoresponse during light On-Off cycle..... **Page S10**
Figure S4. LSV of CN:PDI under AM1.5G and photoresponse during On-Off cycle… **Page S11**
Figure S5. LSV of Exf. CN:PDI under AM1.5G and photoresponse during On-Off...... **Page S11**
Figure S6. LSV of CdS under AM1.5G and photoresponse during On-Off cycle……… **Page S12**
Figure S7. LSV of CdS/CN:PDI under AM1.5G and photoresponse during On-Off… **Page S12**
Figure S8. Photocatalytic dye degradation results using CN and CN:PDI .... **Page S13**
Figure S9. Band-diagram of photoanodes subjected to positive applied bias in Na_2S............ **Page S13**
Table S1. The elemental composition of materials determined using XPS analysis .... **Page S8**
Table S2. Fitting elements of the equivalent circuit obtained from the EIS Nyquist plot... **Page S9**
Table S3. Comparison of photocatalytic activity for benzyl alcohol oxidation .... **Page S14**
Table S4. Comparison of photocatalytic activity for dye degradation .... **Page S15**

1. Experimental section

1.1 Chemical reagents

Pyromellitic dianhydride; PMDA (97%), melamine (99%), dicyandiamide (99%), Cadmium chloride hydrate (98%), thiourea (99%), anhydrous Na_2SO_4 (99%), titanium diisopropoxide (97%), acetic acid (≥99.85%), Rhodamine B; $C_{28}H_{31}ClN_2O_3$ (≥95%) and methylene blue ($C_{16}H_{18}ClN_3S\cdot xH_2O$) were obtained from Sigma Aldrich. Conc. nitric acid, HCl (37%) was procured from Fischer Scientific. All chemicals were used as received without any further purification. HPLC grade solvents and DI water were used throughout the experiments. Conductive Fluorine-doped tin oxide (FTO) glass substrates were purchased from Hartford Tec Glass Company (specifications: TEC 7, resistivity: 6-8 ohm/square, visible

transmittance: 80-82 %, haze: 5%). The FTO glass was cleaned with acetone, methanol and water respectively under sonication for 10 min to remove any organic-inorganic impurities.

2. Synthesis of photocatalytic materials

2.1 Synthesis of bulk graphitic carbon nitride, g-C_3N_4, CN [1]

Bulk carbon nitride was synthesized by thermal annealing of dicyandiamide at 550 ºC in a semi-closed alumina crucible. 10 g of dicyandiamide was heated in an alumina crucible covered with a closed lid with a heating rate of 8 °C min^{-1} up to 300 ºC and 2 °C min^{-1} up to 550 ºC and finally holding the temperature at 550 °C for 4 h. The obtained pale-yellow solid was finely ground into a powder.

2.2 Synthesis of bulk carbon nitride polydiimide polymer (CN:PDI)[2,3]

The precursor melem was synthesized by heating melamine at 425 ºC overnight in an alumina crucible as discussed in the previous report. The afforded yellowish powder was purified by boiling in water and subsequent filtration [4]. Carbon nitride polydiimide polymer was prepared by the solid-state thermal condensation of melem (2,5,8-triamino-s-heptazine) and pyromellitic dianhydride (PMDA). Previous studies revealed that CN/PDI having equimolar amounts of melem and PMDA is most active for photocatalytic reactions. Therefore, we synthesized CN:PDI by thermal annealing of an equimolar mixture of melem and pyromellitic dianhydride. In brief, a mixture of melem (2.0 g) and pyromellitic dianhydride (4.0 g) was heated at a rate of 7 °C/min up to 325 °C for 4 h. The obtained greenish-yellow solid was ground, washed with hot water and dried.

2.3 Synthesis of exfoliated carbon nitride polydiimide polymer (Exf. CN:PDI)[5]

The bulk CN:PDI was transformed into monolayer/few-layered sheets by proton assisted exfoliation with hot HNO_3. Briefly, a 50 mL round bottom (RB) flask was placed in an ice bath with a magnetic stirrer. The RB flask was charged with 0.5 g CN:PDI powder and then 50 mL Conc. HNO_3 (65 wt.%) was slowly

added with stirring. After 15 min stirring the ice bath was removed and the obtained mixture was heated at 80 °C for 3 h (Precaution: The temperature should be maintained below 100 °C). During this step, the color of solution changed to milky demonstrating the exfoliation of CN:PDI sheets. The obtained suspension was diluted with DI water and centrifuged. The obtained CN:PDI sheets were again dispersed in water and centrifuged followed by repeating this step several times until the pH became neutral. The obtained exfoliated CN:PDI sheets were finally dispersed in methanol for further use.

2.4 Synthesis of CdS nanorods

CdS nanorods were synthesized *via* a hydrothermal approach reported earlier with slight modification [6,7]. In brief, 2.5 mM of $CdCl_2{\cdot}2.5H_2O$ (cadmium dichloride hemipentahydrate) and 7.48 mM of NH_2CSNH_2 (thiourea) were mixed via magnetic stirring in 20 mL of ethylenediamine until the solution became transparent. The obtained solution was transferred in a Teflon autoclave and heated in an oven at 170 °C for 36 h. The obtained yellow solid was washed with methanol and DI water several time and dried in a vacuum oven at 80 °C overnight to get the CdS nanorods.

2.5 CN:PDI wrapped CdS Van der Waals heterostructure (CdS/CN:PDI)

For the synthesis of CdS/CN:PDI, 0.5 g of CdS was added to 10 mL of methanol in a vial followed by sonication of the vial for 15 min. To this solution, a suspension of methanolic Exf. CN:PDI was added and stirred for 12 h. After that, the solid was separated by centrifugation, washed with methanol and dried at room temperature.

3. Physicochemical characterization/measurements

The nanoscopic morphology of materials was determined using high-resolution transmission electron microscopy (HR-TEM), acquired on a JEOL JEM-ARM200CF S/TEM operating at an acceleration voltage of 200 keV. For making samples for TEM, a very dilute suspension of CdS/CN:PDI was made in

methanol by ultrasonication and deposited on a 300-mesh lacy carbon-coated copper TEM grid and dried under a solar simulator for 2h. Gatan micrograph software was used for the processing of acquired electronic TEM images in .dm3 format to analyze shape, size and *d* spacing. Electron energy-loss spectroscopy (EELS) line scan was performed on CdS/CN:PDI samples to validate the presence of constituent elements and the wrapping of CN:PDI around the nanorods. The size distribution of exfoliated CN:PDI sheets, CdS and CdS/CN:PDI was determined using dynamic light scattering (DLS) in water using a Malvern Zetasizer. The chemical composition, binding energy and oxidation state of constituting elements of the samples were determined using X-ray photoelectron spectroscopy (XPS) recorded on an Axis-Ultra, Kratos Analytical instrument and a monochromatic Al-Kα source (15 kV, 50 W) and 1486.7 eV photon energy under ultrahigh vacuum ($\sim 10^{-8}$ Torr). The binding energy of all the elements was referenced with respect to the binding energy of the C1s peak of adventitious carbons at ≈ 284.8 eV. The XPS spectra in .vms format were deconvoluted into various peak components using CasaXPS software. The vibrational features specific to various functional groups were determined using Fourier transform infrared (FT-IR) spectroscopy acquired on a Digilab (Varian) FTS 7000 FT-Infrared Spectrophotometer with UMA 600 Microscope equipped with a ZnSe ATR accessory. For the measurement, the samples were deposited on a ZnSe crystal followed by maintaining nitrogen flow using an ATR accessory, and spectra were accumulated by averaging 32 scans in the frequency range of 400–4000 cm^{-1}. The crystalline nature, periodicity and phase structure of the materials were determined using X-ray diffraction (XRD) recorded on a Bruker D8 Discover instrument using Cu-Kα radiation (40 kV, λ = 0.15418 nm) equipped with a LynxEYE 1-dimensional detector. The spectra were recorded in the 2θ range 4–60° with a 0.02° scan size. The absorption profile of the materials in the UV-Vis region was determined using a Perkin Elmer Lambda-1050 UV–Vis-NIR spectrophotometer equipped with an integrating sphere accessory. The UV-Vis spectra of thin films of synthesized materials deposited on a glass slide were recorded in diffuse

reflectance mode. The nature of charge carrier dynamics and the recombination mechanism were studied using steady-state photoluminescence (*ss*PL) spectra recorded on a Varian Cary Eclipse fluorimeter with a xenon lamp excitation source while using a slit width of 5 mm. The Raman active vibrational features of the materials were measured on a Thermo Scientific DXR2 Raman Microscope with a 632 nm laser excitation and an incident power of 10 mW cm^{-2}. The spectra were collected using a 50 µm confocal pinhole apertures slit, a 2 cm^{-1}/CCD pixel element spectral dispersion grating and 60 s accumulation time.

4. Calculation of applied bias photon-to-current efficiency (ABPE)

The performance of photocatalyst and materials/interface behavior under various conditions such as applied bias and illumination wavelength was determined by calculating the diagnostic ABPE [8–10]. The maximum photoconversion efficiency (PCE%) under the conditions of applied bias can be expressed by applied bias photon-to-current efficiency percentage (ABPE%). The ABPE as a function of applied potential on the reversible hydrogen electrode (RHE) scale was calculated by using the following expression:

$$ABPE\ (\%) = [J\ (mA\ cm^{-2}) \cdot \frac{1.23 - V_b}{P(mW\ cm^{-2})}] \cdot 100 \qquad (1)$$

Where J is photocurrent density, V_b applied voltage at the RHE scale and P is the power density of the incident light.

The applied potential vs Ag/AgCl scale was converted to the RHE scale by using the following equation.

$$V_{RHE} = V_{Ag/AgCl} + 0.059\ pH + V^0_{Ag/AgCl} \qquad (2)$$

Where, $V^0_{Ag/AgCl} = 0.197$ V.

The ABPE% for CN, Bulk CN:PDI, Exf. CN:PDI, CdS, CdS/CN:PDI were found to be 0.011, 0.075, 0.137, 0.210 and 0.335% under AM1.5 G irradiation, respectively.

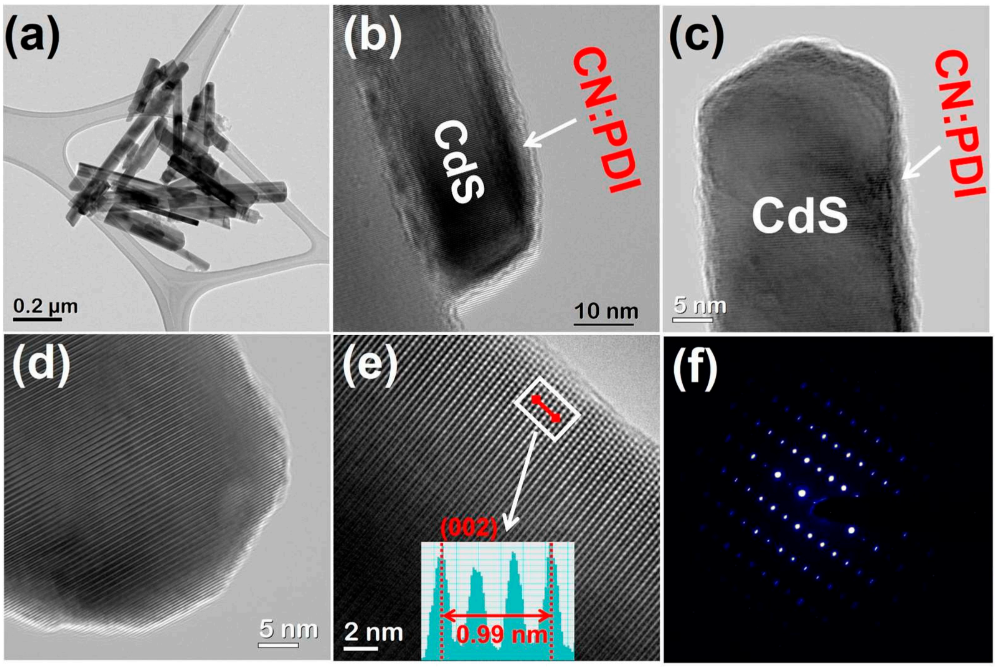

Figure S1. HR-TEM images of CdS/CN:PDI at (a) 0.2 µm scale bar (b–c) 10 nm scale bar showing CN:PDI wrapped CdS nanorods (d) HR-TEM image showing lattice fringes (e) HR-TEM image at 2 nm scale showing the atomic column. Inset showing interplanar d-spacing between atomic dots (f) SAED pattern of CdS/CN:PDI.

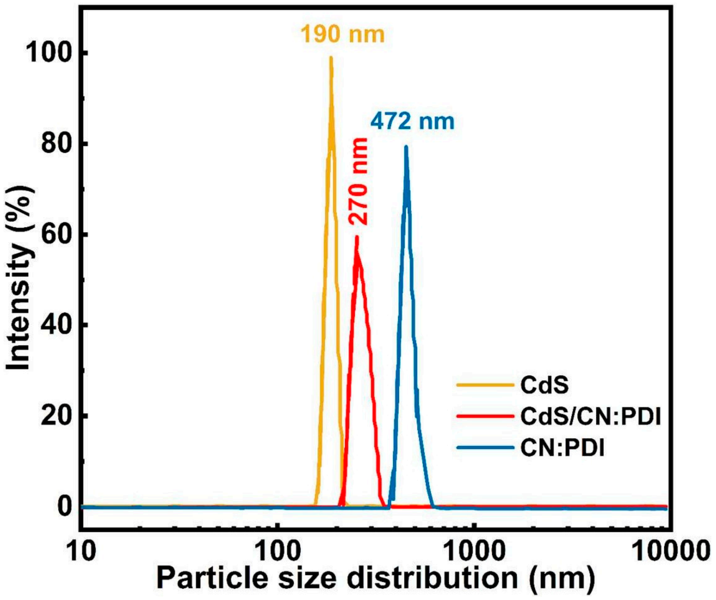

Figure S2. The average particle size distribution of CdS nanorods (yellow), Exf. CN:PDI and CdS/CN:PDI (red) in water as calculated with dynamic light scattering (DLS).

Table S1. The elemental composition of materials determined using XPS analysis.

S.No.	Materials	C (at%)	N (at%)	O (at%)	Cd (at%)	S (at%)
1.	CdS	-	-	33.17	41.16	25.70
2.	CN	44.27	51.46	4.27	-	-
3.	CN:PDI	38.51	44.42	17.07	-	-
4.	CdS/CN:PDI	41.38	7.73	19.95	20.75	10.19

5. Electrochemical impedance spectroscopy

To investigate the electron-diffusion and interfacial charge-transfer properties of the photoelectrode made from the synthesized materials, electrochemical impedance spectroscopy (EIS) was conducted under dark and AM 1.5 G solar simulated light conditions in the 0.1 to 100,000 Hz frequency range and −0.5 V *vs* Ag/AgCl [11,12]. The Nyquist plot shows a semicircle in the high-frequency region and a curved line at the low-frequency region. The Randles equivalent circuit obtained by fitting the experimental data gave various parameters of materials-electrolyte interaction shown in Figure 5h-i and Table S2. In the equivalent circuit, R_S R_{SC} and R_{CT} are the solution resistance, space-charge resistance, and charge-transfer resistance respectively, while C_{sc}, C_H, Q and *n* are space-charge capacitance, Helmholtz capacitance, constant phase element, and coefficient. The obtained values are listed in Table S2. The diameter of the semicircle represents charge transfer resistance between solvent and electrode, and the shorter semicircle represents the exfoliated and heterojunction materials representing low resistance of charge migration from solvent to materials. The value of charge transfer resistance for the samples was obtained in the following order under dark conditions CdS/CN:PDI (4.065) <Exf. CN:PDI (33.37) <melem (92.56) <CN (168.5) <CN:PDI (239.4) <CdS (453.3). From these values, it can be seen that pristine CdS has the highest charge transfer resistance due to low surface area, hydrophobicity and plenty of defect states. Similarly, bulk CN and CN:PDI displayed higher values of charge transfer resistance. However, when bulk CN:PDI was exfoliated into mono- to few-layered sheets the value of R_{CT} was significantly decreased suggesting

better electronic transport on the conjugated monolayer sheets. Interestingly, in CdS/CN:PDI Van der Waals heterojunction, the value of R_{CT} was as low as 4.065 Ω which suggests decreased interfacial charge recombination between the heterojunction electrode and the electrolyte. As expected, under solar light irradiation the charge transfer resistance increased for all the samples, which was corroborated by upward bend bending in the semiconductor.

Table S2. The EIS Nyquist plot fitting parameters to extract various fitting elements of the equivalent circuit under dark and light (AM1.5 G) conditions.

Sample	R_S (Ω)	C_{SC} (F)	R_{SC} (Ω)	C_H (ohm.$s^{-1/2}$)	R_{CT} (Ω)	Q (F.$s^{(-1+n)}$)	n
**Melem light**	15.36	10.97×10^{-9}	52	1007	122.1	71.13×10^{-6}	0.8586
**Melem dark**	15.22	10.6×10^{-9}	50.72	1778	92.56	62.88×10^{-6}	0.8241
**CN light**	38.24	7.023×10^{-9}	135.4	1345	484.5	73.15×10^{-6}	0.8927
**CN dark**	41.57	8.16×10^{-9}	143.7	3790	168.5	40.33×10^{-6}	0.7777
**CN:PDI light**	17.61	9.138×10^{-9}	55.81	1202	97.28	22.16×10^{-6}	0.9997
**CN:PDI dark**	18.53	7.993×10^{-9}	60.08	1546	239.4	63.48×10^{-6}	0.8575
**Exf. CN:PDI light**	18.99	8.853×10^{-9}	69.75	712	290.9	0.171×10^{-3}	0.8142
**Exf CN:PDI dark**	18.64	8.851×10^{-9}	64.77	1066	33.37	42.46×10^{-6}	0.9901
**CdS light**	17.41	9.536×10^{-9}	58.4	1857	350.7	60.79×10^{-6}	0.8602
**CdS dark**	20.21	7.146×10^{-9}	71.69	3160	453.3	46.35×10^{-6}	0.82
**CdS/CN:PDI light**	18.46	9.719×10^{-9}	73.38	-4393	39.15	3.979×10^{-6}	0.8715
**CdS/CN:PDI dark**	18.02	6.911×10^{-9}	74.99	7010	4.065	15.79×10^{-6}	0.9104

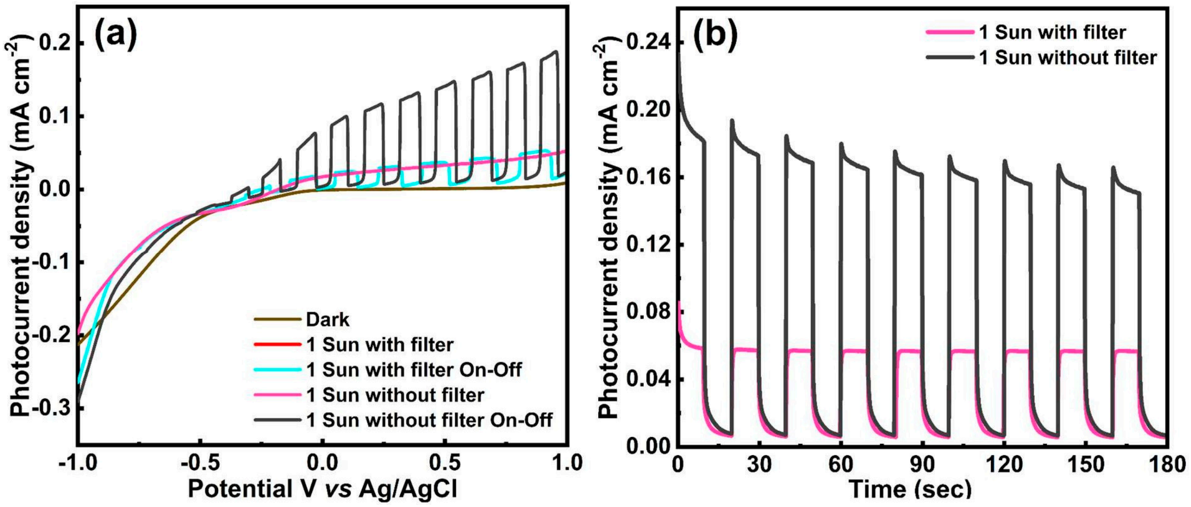

Figure S3. (a) Linear sweep voltammogram (LSV) of CN showing photocurrent density *vs* applied potential under AM1.5G light irradiation without filter (100 mW cm^{-2}) and AM1.5G light irradiation with 420 nm cut-off filter (b) Photocurrent response as a function of time (*i-t*) during light On-Off cycle at +0.6 V applied potential for CN under solar simulated AM1.5G light irradiation without filter (100 mW cm^{-2}) and AM1.5G light irradiation with 420 nm cut-off filter.

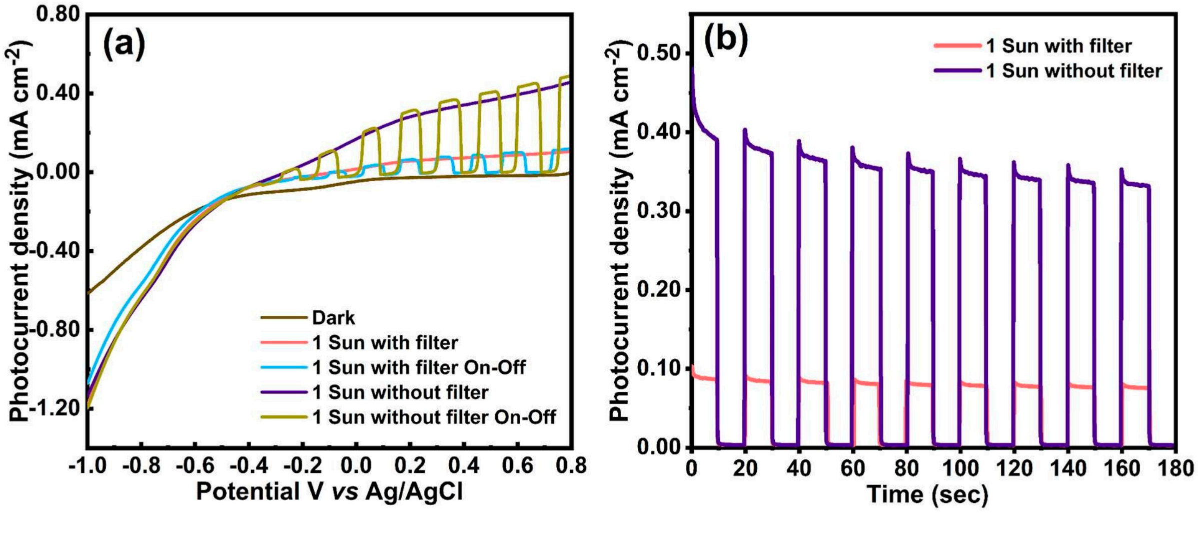

Figure S4. (a) Linear sweep voltammogram (LSV) of CN:PDI showing photocurrent density *vs* applied potential under AM1.5G light irradiation without filter (100 mW cm^{-2}) and AM1.5G light irradiation with 420 nm cut-off filter (b) Photocurrent response as a function of time (*i-t*) during light On-Off cycle at +0.6 V applied potential for CN:PDI under

solar simulated AM1.5G light irradiation without filter (100 mW cm^{-2}) and AM1.5G light irradiation with 420 nm cut-off filter.

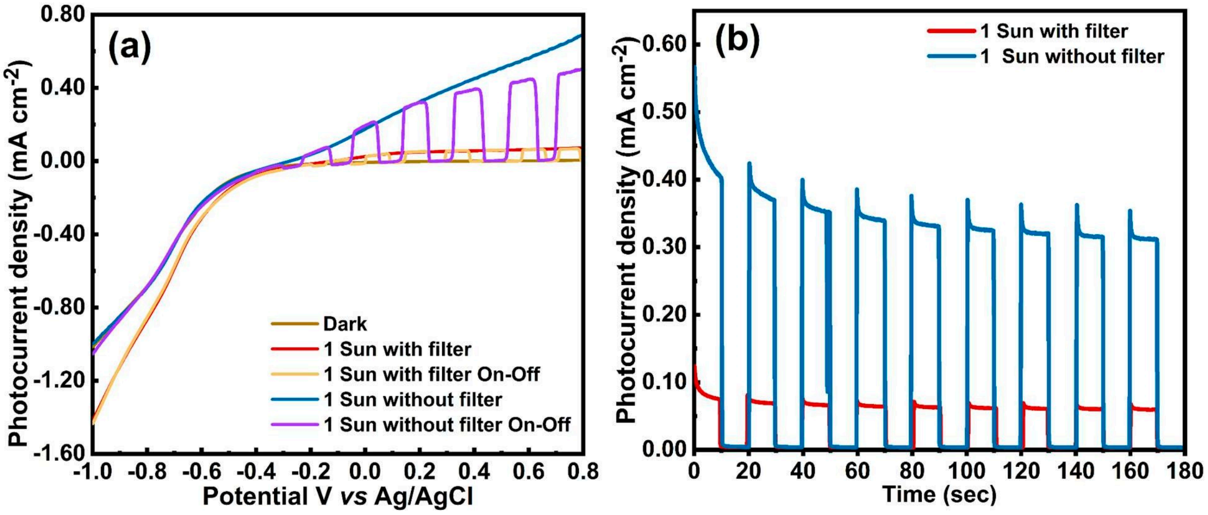

Figure S5. (a) Linear sweep voltammogram (LSV) of Exf. CN:PDI showing photocurrent density *vs* applied potential under AM1.5G light irradiation without filter (100 mW cm^{-2}) and AM1.5G light irradiation with 420 nm cut-off filter (b) Photocurrent response as a function of time (*i-t*) during light On-Off cycle at +0.6 V applied potential for Exf. CN:PDI under solar simulated AM1.5G light irradiation without filter (100 mW cm^{-2}) and AM1.5G light irradiation with 420 nm cut-off filter.

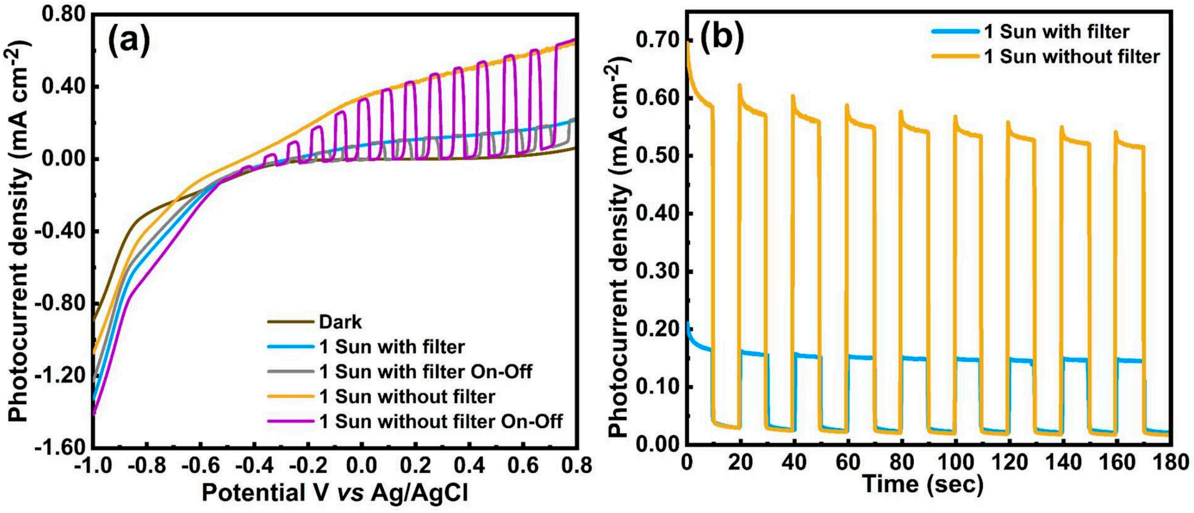

Figure S6. (a) Linear sweep voltammogram (LSV) of CdS showing photocurrent density *vs* applied potential under AM1.5G light irradiation without filter (100 mW cm^{-2}) and AM1.5G light irradiation with 420 nm cut-off filter (b)

Photocurrent response as a function of time (*i-t*) during light On-Off cycle at +0.6 V applied potential for CdS under solar simulated AM1.5G light irradiation without filter (100 mW cm^{-2}) and AM1.5G light irradiation with 420 nm cut-off filter.

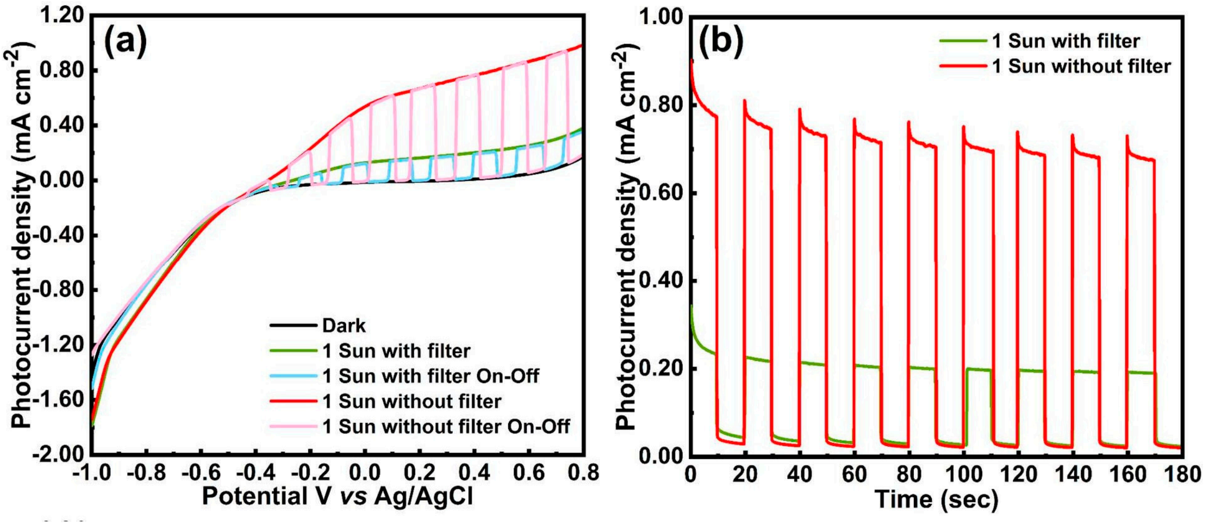

Figure S7. (a) Linear sweep voltammogram (LSV) of CdS/CN:PDI showing photocurrent density *vs* applied potential under AM1.5G light irradiation without filter (100 mW cm^{-2}) and AM1.5G light irradiation with 420 nm cut-off filter (b) Photocurrent response as a function of time (*i-t*) during light On-Off cycle at +0.6 V applied potential for CdS/CN:PDI under solar simulated AM1.5G light irradiation without filter (100 mW cm^{-2}) and AM1.5G light irradiation with 420 nm cut-off filter.

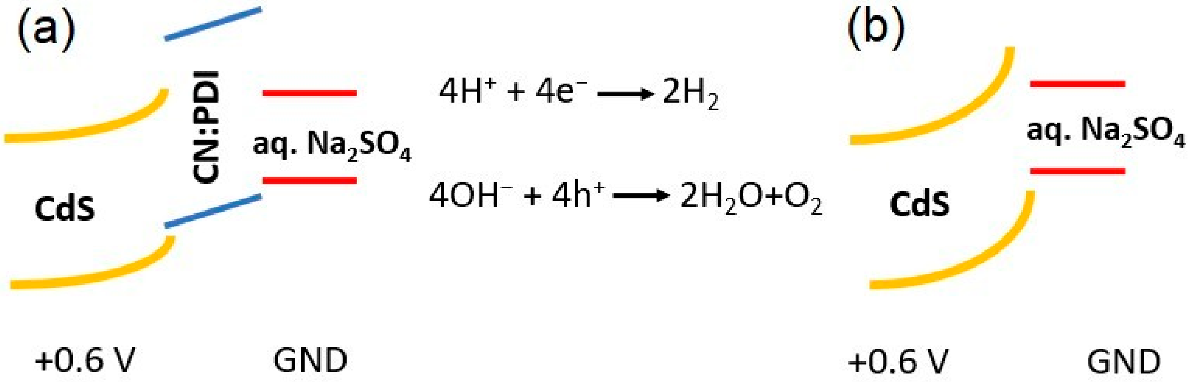

Figure S8. Band-diagram of photoanodes subjected to positive applied bias in aqueous sodium sulfate electrolyte (a) CdS/CN:PDI and (b) CdS

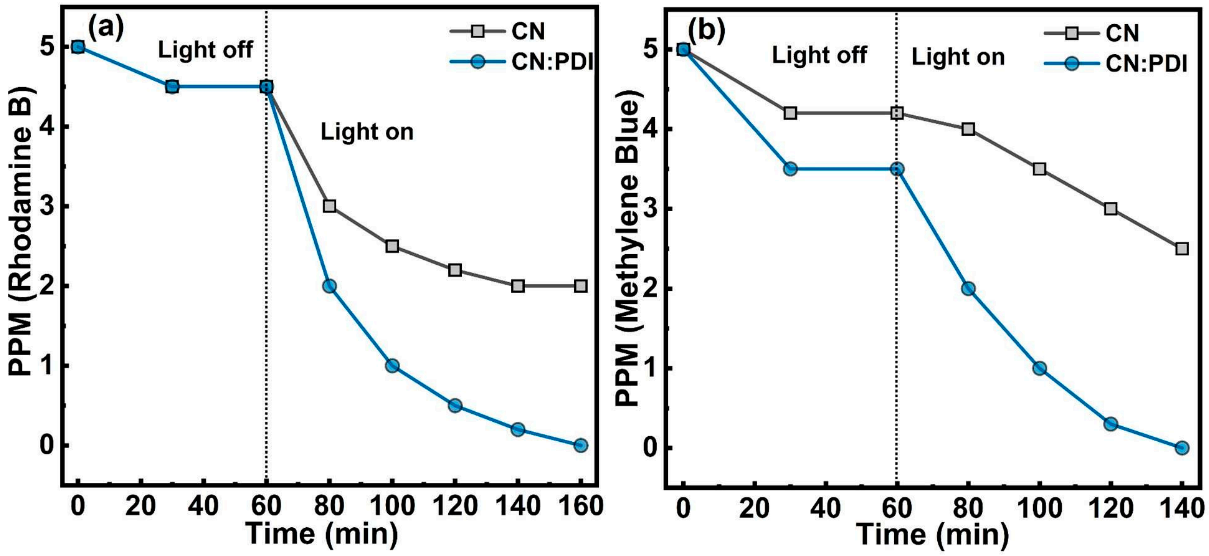

Figure S9. Photocatalytic dye degradation results using CN and CN:PDI photocatalysts (a) degradation of RhB (b) degradation of MB.

Table S3. Comparison of photocatalytic activity of CdS/CN:PDI for dye degradation with state-of-the-art catalysts

S No.	Catalyst	Dye	Catalysts concentration (mg/100 mL)	Light Source (power density- mW cm^{-2})	Irradiation time (min)	Degradation Efficiency	Ref.
**1.**	$Fe_3O_4@MnO_2$	Rhodamine B	10	- (-)	120	75 %	[13]
**2.**	$CoW_{12}O_{40}$ /$BiVO_4$	Methylene blue	50	300 W Xe (-)	150	97%	[14]
**3.**	CdS NPs	Methylene blue	40	- (-)	180	97%	[15]
**4.**	HfO_2 doped TiO_2	Methylene blue	40	300 W Xe (100 mW cm^{-2})	50	>90%	[16]
**5.**	ZnO-TiO_2	Methyl orange	100	300 W Xe (100 mW cm^{-2})	180	92%	[17]
**6.**	$CuCr_2O_4$/$BiOBr$	Rhodamine B	41.7	50 W COOL	15	96%	[18]

				LED lamp (-)			
**7.**	ZnS QDs/Co_3O_4-coupled g-C_3N_4	Rhodamine B	50	- (-)	180	93%	[19]
**8.**	Ce doped ZnO nanowires	Methylene blue Rhodamine B	100	50 W COOL LED lamp	20	96.6% 77.7%	[20]
**9.**	g-C_3N_4/Ag_2WO_4/Bi_2S_3	Congo red	50	140 W LED lamps (111 mW cm^{-2})	60	98%	[21]
**10.**	Nb_2O_5/BRGO	Crystal violet	40	300 W Xe (-)	90	98%	[22]
**11.**	ZnS/PbS	Methylene blue,	-	(500 mWatt cm^{-2})	120 min	91.2%	[23]
**12.**	CuS/ZnS and CuS/CdS	Methylene blue	40 mg	300 W Xe (λ>420 nm) (750 mW cm^{-2})	30 min	95%	[24]
**13.**	Cu_2O@$Zn(OH)_2$	Methyl orange	37.5 mg	300 W Xe (λ>400 nm) (100)	150 min	96.6%	[25]
**14.**	Ag_3PO_4/Bi_2SiO_5	Rhodamine B Methylene blue	100 mg	70 W Xe (-)	90 min	92.86% 95.56%	[26]
**15.**	Activated C/MgO	Rhodamine -B	100 mg	150 W Xe (λ>420 nm) (100) (-)	40 min	99%	[27]
**16.**	Au@Polydopamine-Ti_3C_2	Methylene blue	0.05 mg	- (-)	4 min	100%	[28]
17	CdS/CN:PDI	Methylene blue Rhodamine - B	20 mg	300 W Xe (λ>420 nm) (100)	80 min	100%	This work

Table S4. Comparison of photocatalytic activity of CdS/CN:PDI for benzyl alcohol oxidation with state-of-the-art catalysts.

S.No.	Catalysts	Catalyst Amount	Reaction conditions	O_2	Light source	Time (h)	Yield (%)	Ref.
**1.**	$CsPbBr_3$/P25	25 mg	0.1M BA+ toluene	1bar O_2	300 W Xe (λ>420 nm)	60	87	[29]
**2.**	Au_9–Pd_1/LDH	24 mg	0.1 mM BA+BTF	Pure O_2	300 W Xe (λ>400 nm)	5	91.1	[30]
**3.**	Bi_2MoO_6	16 mg	0.1 mM BA+BTF	Pure O_2	300 W Xe (λ>360 nm)	4	38.2	[31]
**4.**	$Bi_4Ti_3O_{12}$	10 mg	0.1 mM BA +BTF	Pure O_2	150 W Xe (λ >350 nm)	5	35.5	[32]

**5.**	$SnS/g\text{-}C_3N_4$	60 mg	0.2 mM BA+ACN	Pure O_2	300 W Xe (λ>420 nm)	6	99	[33]
**6.**	$Pd/H_2Ti_3O_7$	0.1 g	10 mL BA	Pure O_2	300 W Xe (λ>420 nm)	6	89	[34]
**7.**	$CsPbX_3/W_{18}O_{49}$	0.01 g	0.04 mol/L BA	Pure O_2	150 W Xe (λ>420 nm)	7	50	[35]
**8.**	$Ru/g\text{-}C_3N_{4-x}$	5 mg	0.3 mL BA+DI W	N_2	300 W Xe (λ>320 nm)	3	11.50	[36]
**9.**	$AgBr@Ag@TiO_2$	50 mg	104 μL BA+ACN	1bar O_2	300 W Xe (λ>420 nm)	8	>99	[37]
**10.**	Ni (1%)-$OTiO_2$	80 mg	0.5 mM BA+BTF	1atm O_2	300 W Xe (λ>420 nm)	1	86	[38]
**11.**	$W_{18}O_{49}/ZnIn_2S_4$	40 mg	0.2 mM BA+BTF	1 bar O_2	300 W Xe (λ>420 nm)	3	>99	[39]
**12.**	$BiOBr/Bi_2WO_6$	20 mg	20 μL BA+BTF	Pure O_2	300 W Xe (λ>420 nm)	4	30.9	[40]
**13.**	(POM)-$ZnIn_2S_4$	5 mg	0.02 mol L^{-1} BA	Ar gas	300 W Xe (λ>420 nm)	5	100	[41]
**14.**	$NVs/g\text{-}C_3N_4$	30 mg	20 μM BA+Hexane	Pure O_2	300 W Xe (λ>420 nm)	4	70%	[42]
**15.**	CdS/CN:PDI	50 mg	1.0 mM BA+ ACN	Air	300 W Xe (λ>420 nm)	12	90%	This work

BTF: Benzotrifluoride ACN: Acetonitrile
